# Conservation of wildlife populations: factoring in incremental disturbance

**DOI:** 10.1002/ece3.3015

**Published:** 2017-05-07

**Authors:** Abbie Stewart, Petr E. Komers

**Affiliations:** ^1^ MSES Inc.CalgaryABCanada

**Keywords:** conservation, disturbance, land cover, moose, rate, threshold, wolves

## Abstract

Progressive anthropogenic disturbance can alter ecosystem organization potentially causing shifts from one stable state to another. This potential for ecosystem shifts must be considered when establishing targets and objectives for conservation. We ask whether a predator–prey system response to incremental anthropogenic disturbance might shift along a disturbance gradient and, if it does, whether any disturbance thresholds are evident for this system. Development of linear corridors in forested areas increases wolf predation effectiveness, while high density of development provides a safe‐haven for their prey. If wolves limit moose population growth, then wolves and moose should respond inversely to land cover disturbance. Using general linear model analysis, we test how the rate of change in moose (*Alces alces*) density and wolf (*Canis lupus*) harvest density are influenced by the rate of change in land cover and proportion of land cover disturbed within a 300,000 km^2^ area in the boreal forest of Alberta, Canada. Using logistic regression, we test how the direction of change in moose density is influenced by measures of land cover change. In response to incremental land cover disturbance, moose declines occurred where <43% of land cover was disturbed; in such landscapes, there were high rates of increase in linear disturbance and wolf density increased. By contrast, moose increases occurred where >43% of land cover was disturbed and wolf density declined. Wolves and moose appeared to respond inversely to incremental disturbance with the balance between moose decline and wolf increase shifting at about 43% of land cover disturbed. Conservation decisions require quantification of disturbance rates and their relationships to predator–prey systems because ecosystem responses to anthropogenic disturbance shift across disturbance gradients.

## 1 Introduction

The large‐scale conversion of forest ecosystems to man‐made disturbances can alter the ecosystem organization so that it shifts from one set of processes to another (Scheffer, Carpenter, Foley, Folkes, & Walker, 2001). Shifts in ecosystems can be observed in altered abiotic conditions, animal and vegetation community interactions, or changes in the dominant vegetation type and wildlife species; they can gradually or abruptly lead to an alternative stable state which may limit future conservation options (Groffman et al., 2006; Margules & Pressey, 2000; Scheffer et al., 2001). For conservation to be effective, Noss et al. (2012) suggested that 50% of a region should be managed with conservation as the primary global target in general. The International Boreal Conservation Science Panel (2013) recommended the same threshold for North America's boreal forest in particular.

In large regions of western Canadian jurisdictions, the anthropogenic conversion of boreal forest advances at one of the highest rates in the world and the majority of the forest in the province of Alberta will soon surpass or has already surpassed the 50% threshold (Komers & Stanojevic, 2013). The rapid pace of development in Alberta is largely driven by oil and gas extraction in the oil sand deposit area (Government of Alberta 2012). The Alberta boreal forest covers about 330,000 km^2^, of which 140,000 km^2^ covers the oil sand deposit area (Government of Alberta 2011).

Anthropogenic conversion of the boreal forest has been shown to alter the distribution of predators and their prey. In the boreal forests of western Canada, wolf (*Canis lupus*) predation is one of the main drivers of both abundance and movement of moose (*Alces alces*), one of their main prey (Ballard, Whitman, & Gardner, 1987; Kuzyk, 2002; Latombe, Fortin, & Parrott, 2014). In turn, wolf movement and distribution are facilitated by the development of anthropogenic linear corridors (Latham, Latham, Boyce, & Boutin, 2011; Whittington, St. Clair, & Mercer, 2005). If land cover change, such as increased density of linear corridors, results in increased predation effectiveness, then we would expect wolves and moose to respond inversely to anthropogenic disturbance and the moose populations should decline where linear disturbance facilitates predation. By contrast, ungulate prey, such as moose, appears to increase in landscapes with a high density of development (Maier et al., 2005; Schneider & Wasel, 2000; Torres, Carvalho, Panzacchi, Linnell, & Fonseca, 2011). This is probably because predators are absent from such landscapes and ungulates are relatively safe from predation in the vicinity of human activity (Hebblewhite et al., 2005; Muhly, Semeniuk, Massolo, Hickman, & Musiani, 2011). In addition, forage may be more available in highly fragmented areas (Schneider & Wasel, 2000). This suggests that linear disturbance should facilitate predation up to a certain density of disturbance, but should hinder predation where disturbance is high. Indeed, Whittington et al. (2005) found that wolves use linear corridors up to about 1.0 km/km^2^ but avoid them where the density of linear corridors is higher, and Dickie, Serrouya, McNay, and Boutin (2016) showed that linear corridors improve the search rate of wolves while hunting in forested areas. Messier (1991, 1994) documented the strong effect that wolf predation has on moose densities, showing that wolf predation not only reduces moose densities, but can keep them at low levels, even if ample forage exists for the moose. Messier (1991, 1994) also showed that under high wolf densities, moose population growth is limited, while under low wolf densities, moose can reach carrying capacity at which point they compete for food. If wolf predation increases with incremental disturbance up to a certain density of disturbance and then decreases, we would expect the moose populations to show the inverse relationship. We would further expect that the balance between wolf increase and moose decline would shift somewhere along the disturbance gradient. In order to test these predictions, information is required on how the rate of change in land cover relates to the rate of change in moose populations and how these changes are related to the presence of wolves.

Here, we ask whether the wolf–moose system response to incremental anthropogenic disturbance might shift along the disturbance gradient and if it does, whether our results support the recommendation that 50% of a region should be set aside for conservation of this system. Specifically, we test the relationships between the rate of land cover change and the change in growth rates of moose populations and we relate these changes to wolf abundance.

## 2 Methods

### 2.1 Study design

We measured land cover change in the boreal forest of Alberta in landscapes representing almost the entire disturbance gradient from 7% to 97% land cover disturbed (0.04–1.32 km/km^2^ of linear disturbance), using a satellite image‐based change detection analysis. Three time periods from 1992 to 2008 were selected for the calculation of a rate of conversion of land cover. The time periods were selected based on the best availability of cloud‐free images. As the effects of disturbances may extend into undisturbed habitats (Ries, Fletcher, Battin, & Sisk, 2004), in addition to calculating the direct change in land cover, we also apply a zone of influence (ZOI) around each footprint and each linear feature. We used publicly available data from government moose surveys and wolf harvesting records to evaluate changes in wolf and moose densities in relation to land cover changes.

### 2.2 Study area

**Figure 1:**
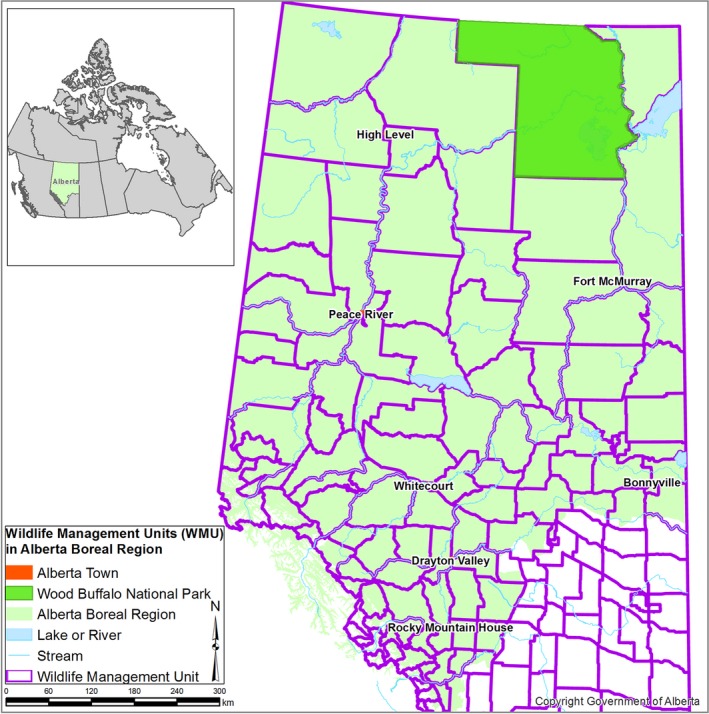
Alberta boreal forest study area showing Northern Boreal Wildlife Management Units

The study area includes the boreal forest of Alberta, excluding National Parks or water bodies, (see Komers and Stanojevic (2013)) (Figure 1). Vegetation in the Boreal Forest Natural Region (BFNR) broadly consists of deciduous, mixedwood, and coniferous forests interspersed with extensive wetlands (Natural Regions Committee 2006). The most common deciduous species are aspen (*Populus tremuloides*) and balsam poplar (*Populus balsamifera*) and the dominant conifers are white spruce (*Picea glauca*), black spruce (*Picea mariana)*, and jack pine (*Pinus banksiana)*. Wetlands are primarily black spruce, shrub, or sedge fens. The BFNR contains an abundance of natural resources which are undergoing rapid industrial development including oil sands, coal, metal, and mineral mining, oil and gas activity, timber operations, agriculture, and urbanization (Government of Alberta 2012). These activities all contribute to the loss of boreal forest cover while associated infrastructure such as access roads, seismic lines, and pipelines have contributed to the fragmentation of the boreal forest. With established reserves still remaining, growth in many of these resource sectors is likely to continue (Government of Alberta 2012).

### 2.3 Satellite imagery classification

Methods for satellite imagery analysis, unsupervised classification, detecting change of land cover, digitizing of visible linear corridors, and accuracy assessment followed those described in Komers and Stanojevic (2013). We used data processing based on the image algebra method to complete the change analysis (Singh, 1989; Stanojevic, Lee, & Gysbers, 2006; Wickware & Howarth, 1981). The image algebra method allows for a mathematical (algebraic) comparison of two spatially overlapping datasets (pixel values) (Jensen, 2005; Potapov, Turubanova, & Hansen, 2011). This method has been reported as one of the most accurate change detection algorithms (Jensen, 1981; Jensen & Toll, 1982) with an overall accuracy near 90% reported for standardized differencing (Bauer et al., 1994) and for the Fort McMurray region (Alsadat, Das, & El‐sheimy, 2010). Landsat Program (1988–2009) images were used for the changes analysis because of their medium resolution (pixel size 28.5 × 28.5 m) and temporal availability (NASA 2013). The images used for this study were from 1990 (±2 years; Landsat‐5), 2000 (±2 years; Landsat‐7), and 2008 (±1 year; Landsat‐5). Anthropogenic disturbances were mapped and changes quantified between 1992/2002 and 2002/2008 to produce a rate of change in disturbed land cover for each Wildlife Management Unit (WMU) in the boreal forest of Alberta. We deviated in one aspect from the methods used by Komers and Stanojevic (2013): Cutblocks present in 1992 were classified as undisturbed land cover. These cutblocks have an unknown initial date of forest harvest and, given that moose forage is typically abundant in cutblocks 7–10 years old (Fischer & Wilkinson, 2005), we made the assumption that these cutblocks provide some moose forage. However, cutblocks identified in 2002 and 2008 were classified as disturbed land cover since harvest occurred sometime within the previous 6–10 years and vegetation likely had insufficient time to recover.

### 2.4 Detecting moose habitat change

We defined “moose habitat” as vegetation types that are used more frequently than expected based on availability. Stewart, Komers, and Bender (2010) described moose habitat as shrubland, deciduous, and water cover classes. These habitat relationships were also observed in other studies of moose habitat use (Cairns & Telfer, 1980; Peek, 1997). We classified moose habitat, as defined above, using the 2002 Alberta Ground Cover Classification (AGCC) and performed a change analysis for moose habitat using the methods described above under “Satellite imagery classification” (Komers & Stanojevic, 2013). The rate of change in disturbed land cover is significantly, negatively correlated (Pearson, *N* = 20, *R* = −.89, *p* < .005) with rate of change in moose habitat. When the rate of land cover disturbance is high, there is a corresponding high loss in moose habitat. Therefore, we only present results for the rate of change in land cover with the understanding that this measure also incorporates changes in moose habitat.

### 2.5 Zone of influence

The effects of disturbances may extend into undisturbed habitats (Ries et al., 2004), for a distance known as a zone of influence (ZOI). The ZOI varies by species, habitat type, and disturbance type. To evaluate the moose ZOI, we reviewed studies on habitat use near common sources of disturbance. Moose presence near roads was reduced within approximately 800–950 m (Rolley & Keith, 1980) and within 500 m (Laurian et al., 2008). Moose suffer higher mortality from wolf predation near trails (median distance of kills was 209 m, compared to random sites at 470 m, Kunkel & Pletscher, 2000). Moose presence near agricultural fields was reduced within approximately 400 m (Rolley & Keith, 1980). Thus, we used a ZOI of 250 m, which appears to be conservatively narrow given the effect distances we reviewed.

### 2.6 Moose density and land cover data

Moose densities were estimated using aerial ungulate surveys (AUS) using random stratified block designs (ACA 2012). We compiled estimates of moose density (# individuals/km^2^) for 20 boreal wildlife management units (WMU) between 1992 and 2011 from AUS reports and from regional wildlife biologists (moose density estimates ranged from 0.04 to 0.72 moose/km^2^). For those WMUs with moose density estimates for at least 2 years, we calculated an average yearly rate of change in moose density using the earliest and latest year of data availability in each WMU.

We calculated the amount of disturbed land cover (km^2^) for 1992, 2002, and 2008 for each boreal WMU; however, information on moose density is not always available for each of these 3 years for each WMU. To ensure rates of change in disturbed land cover and moose density were calculated between corresponding years, the percent of disturbed land cover was estimated for each year that corresponded to moose data availability. This estimate of disturbed land cover was determined by extrapolating from trend line plots of land cover change between 1992 and 2008 for each WMU that had an associated rate of change in moose density. We also calculated linear feature density (km/km^2^) for 1992, 2002, and 2008 for each WMU and used the same procedure to determine rates of change in the density of linear features for each WMU.

### 2.7 Predation risk

According to Robichaud and Boyce (2010), wolf harvest (total harvest, number of traplines with harvest, and average number of wolves taken/trapline) in Alberta increased relative to wolf population increases. Wolf harvest increased over time despite apparently stable or decreasing trends in trapper effort which suggests an increasing provincial wolf population (Robichaud & Boyce, 2010). Therefore, we used wolf harvest data to evaluate how wolf populations change in relation to land cover disturbance. The total harvest of gray wolf on Registered Fur Management Areas from 1992 to 2011 in Alberta was provided by AESRD (AESRD 2014). The total wolf harvested per WMU was divided by WMU area to provide a wolf harvest density (#wolves harvested/km^2^) in each WMU.

### 2.8 Statistical analysis

We calculated average annual rate of change in moose density (%), average annual rate of change in percent disturbed land cover (%), and average annual rate of change in linear feature density (%) for each boreal WMU. We also determined moose density, the amount of disturbed land cover, and linear feature density in the years that moose data were first available in each WMU. These latter variables are used to understand the starting conditions present in each WMU (i.e., the conditions that may influence subsequent rates and directions of change in measured parameters). The rate of change in disturbed land cover was significantly correlated with the rate of change in linear feature density (Pearson, *N* = 20, *R* = .85, *p* < .005). Therefore, we developed 16 regression models designed to manage collinearity issues and help us understand how land cover disturbance might influence the rate of change in moose density (Table 1). Moose density was included as a covariate in some models. Rate of change in moose density was regressed against various combinations of variables using a general linear model (GLM) analysis. Candidate models were determined using Akaike's Information Criterion adjusted for small sample sizes (AICc), a metric that provides the relative likelihood of any model given the available data (Burnham & Anderson, 1998).

**Table 1:** Suite of models used to evaluate whether the rate of change in moose density is related to measures of land cover disturbance and change

Model identification	Model variables
1	Amount of disturbed land cover + Rate of change in disturbed land cover + Amount of disturbed land cover *Rate of change in disturbed land cover + Moose density
2	Amount of disturbed land cover + Rate of change in disturbed land cover + Amount of disturbed land cover*Rate of change in disturbed land cover
3	Amount of disturbed land cover + Rate of change in disturbed land cover
4	Amount of disturbed land cover + Rate of change in disturbed land cover + Moose density
5	Amount of disturbed land cover
6	Amount of disturbed land cover + Moose density
7	Rate of change in disturbed land cover
8	Rate of change in disturbed land cover + Moose density
9	Linear feature density + Rate of change in linear feature density + Linear feature density*Rate of change in linear feature density + Moose density
10	Linear Feature Density + Rate of change in linear feature density + Linear feature density*Rate of change in linear feature density
11	Linear feature density + Rate of change in linear feature density
12	Linear feature density + Rate of change in linear feature density + Moose density
13	Linear Feature density
14	Linear feature density + Moose density
15	Rate of change in linear feature density
16	Rate of change in linear feature density + Moose density
17	Linear feature density + Linear feature density*Linear feature density

In order to understand how landscape disturbance might influence wolf populations, wolf harvest density was analyzed using the same methods and suite of models developed to analyze rate of change in moose density (Table 1). Wolf harvest density was square root transformed for all analyses. Scatterplots of wolf harvest density and linear feature density suggested that these variables may be nonlinearly related. Therefore, we added the relevant quadratic model to the suite of models for comparison (Model #17: Linear feature density + Linear feature density*Linear feature density) for both moose and wolves.

## 3 Results

### 3.1 Moose

**Figure 2:**
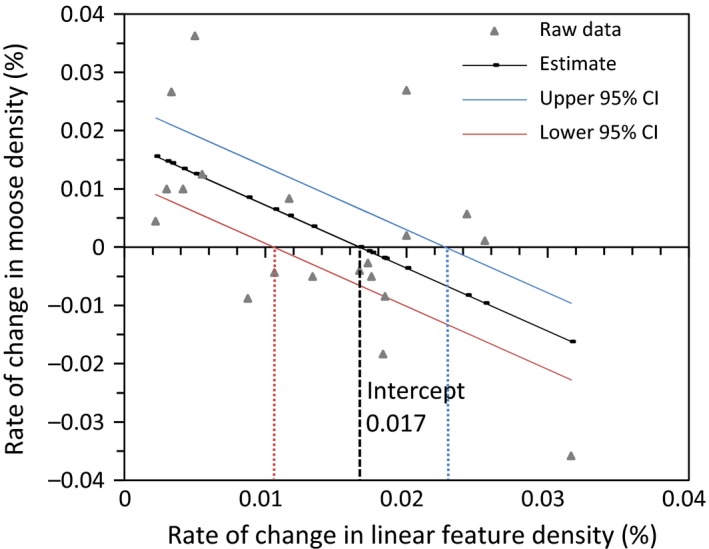
Rate of change in moose density in relation to rate of change in linear feature density per Wildlife Management Unit (WMU) in the Alberta boreal forest. The rate of change in moose density switched from increasing to decreasing at 0.017% rate of change in linear feature density

**Figure 3:**
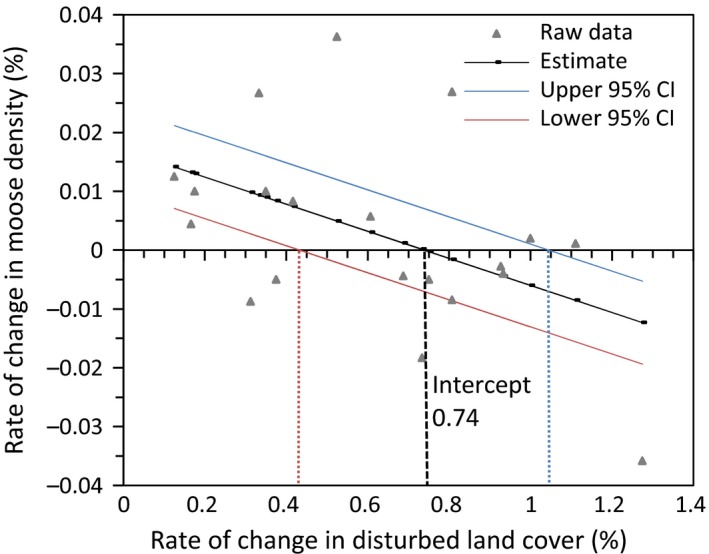
Rate of change in moose density in relation to rate of change in disturbed land cover per WMU in the Alberta boreal forest. The rate of change in moose density switched from increasing to decreasing at 0.74% rate of change in disturbed land cover

A total of 17 candidate models were ranked using AICc. The top three models are listed in Table 2. The top model (#15) indicates that the rate of change in moose density decreased linearly with increasing rate of change in linear feature density (Table 2; Figure 2). The next best model (#7) indicates that the rate of change in moose density decreased linearly with increasing rate of change in disturbed land cover (Table 2; Figure 3). Although this model has a **∆** AIC over 5, the correlation between rate of change in linear feature density and rate of change in disturbed land cover suggests that land cover disturbance may also influence rate of change in moose density. Moose density increased in WMUs with little land cover change, but decreased in WMUs with >0.017% (95% CI [0.01, 0.023]) rate of change in linear feature density and >0.74% (95% CI [0.45, 1.05]) rate of change in disturbed land cover change per year (Figures 2 and 3).

**Table 2:** Top models for describing the influence of land cover disturbance on the rate of change in moose density in the boreal forest of Alberta. Statistics include overall calculated probability (Model *p*), the number of parameters used in each model (*K*), *R*‐squared (*R* ^2^), individual variable standardized coefficient (Std. Coefficient), coefficient standard error (*SE*), coefficient calculated probability (Variable *p*), and change in AIC for small sample sizes (∆ AICc). The number of samples (*N*) is 20 for all models. Model Identification is described in Table 1

Model identification	Model *p*	*K*	*R* ^2^	Variable name	Std. coefficient	*SE*	Variable *p*	∆ AICc
15	.009	3	.319	Rate of change in linear feature density	−0.565	0.371	.009	0
7	.037	3	.220	Rate of change in disturbed land cover	−0.469	0.010	.037	5.754
13	.143	3	.115	Linear feature density	−0.339	0.013	.143	5.754

### 3.2 Predation risk

**Figure 4:**
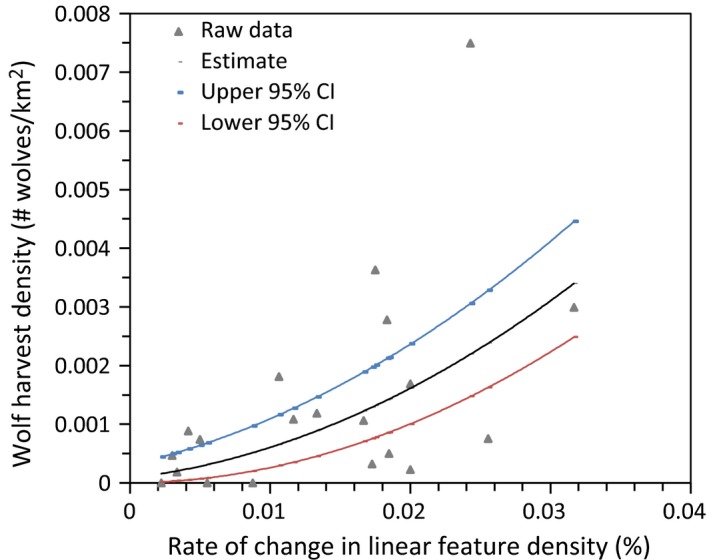
Wolf harvest density in relation to the rate of change in linear feature density per WMU in the Alberta boreal forest

**Figure 5:**
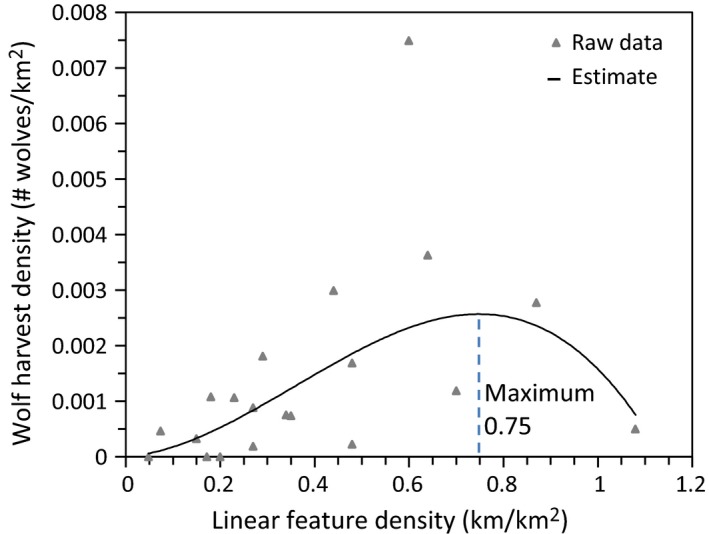
Wolf harvest density in relation to the linear feature density per WMU in the Alberta boreal forest. Wolf harvest density peaks at a linear feature density of 0.75 km/km^2^

A total of 17 candidate models that describe how land cover change may influence wolf harvest density were ranked using AICc. The top three models are listed in Table 3. All other models had a **∆** AICc >2. The top model (#11) indicates that wolf harvest density increased with increasing rate of change in linear density (Table 3; Figure 4), as does another high‐ranked model (#15). Linear feature density was also included in model #11, but the variable itself was not significant. The next best model (#17) indicates that wolf harvest density had a quadratic relationship with linear feature density (Table 3; Figure 5). Wolf harvest density increased and peaked at a linear feature density of 0.75 km/km^2^) and subsequently decreased as linear feature density continued to increase.

**Table 3:** Top models for describing the influence of land cover disturbance on wolf harvest density in the boreal forest of Alberta. Statistics include overall calculated probability (Model *p*), the number of parameters used in each model (*K*), *R*‐squared (*R* ^2^), individual variable standardized coefficient (Std. Coefficient), coefficient standard error (*SE*), coefficient calculated probability (Variable *p*), and change in AIC for small sample sizes (∆ AICc). The number of samples (*N*) is 20 for all models. Model identification is described in Table 1

Model identification	Model *p*	*K*	*R* ^2^	Variable name	Std. coefficient	*SE*	Variable *p*	∆ AICc
11	.011	4	.413	Linear feature density	0.254	0.017	.252	0
				Rate of change in linear feature density	0.477	0.553	.040	
17	.004	4	.472	Linear feature density	2.117	0.050	.003	0.026
				Linear feature density*Linear feature density	−1.694	0.046	.015	
15	.005	3	.364	Rate of change in linear feature density	0.603	0.484	.005	0.505

### 3.3 Increasing versus decreasing populations of moose

Given that the rate of change in moose density shifts from a positive rate of change to a negative rate of change along a land cover disturbance gradient (see Figures 2 and 3), we wanted to evaluate how the probability of having a positive rate of change of moose density is influenced by land cover disturbance. We performed logistic regression using the same suite of models developed to analyze rate of change in moose density (Table 1), but with a binary response variable identifying each WMU as having either a positive or negative moose rate of change. A total of 17 candidate models were ranked using AICc. The top three models are listed in Table 4. All other models had a **∆** AICc >2. The top model (#6) indicates that the amount of disturbed land cover present and moose density influenced the probability of having a positive rate of change in moose density. The next best model (#5) also indicates that the amount of disturbed land cover influenced the probability of having a positive rate of change in moose density. The average amount of disturbed land cover (%) is higher in those WMUs with increasing moose density (mean = 58.55% disturbed land cover; *SE* = 7.43) than in those WMUs with decreasing moose density (mean = 36.52% disturbed land cover; *SE* = 6.84).

**Table 4:** Top models for describing the influence of land cover disturbance on the probability of having a positive rate of change in moose density in the boreal forest of Alberta. Statistics include the overall calculated probability (Model *p*), number of parameters used in each model (*K*), Mcfadden's pseudo *R*‐squared (*R* ^2^), individual variable coefficient (Coefficient), *p* coefficient standard error (*SE*), coefficient calculated probability (Variable *p*), and change in AIC for small sample sizes (∆ AICc). The number of samples (*N*) is 20 for all models. Model identification is described in Table 1

Model identification	Model *p*	*K*	*R* ^2^	Variable name	Coefficient	*SE*	Variable *p*	∆ AICc
6	.021	4	.281	Amount of disturbed land cover	0.085	0.046	.064	0
				Moose density	−10.430	7.998	.192	
5	.036	3	.159	Amount of disturbed land cover	0.044	0.023	.063	0.502
7	.087	3	.107	Rate of change in disturbed land cover	−2.547	1.603	.112	1.948

### 3.4 Threshold in land cover disturbance

**Figure 6:**
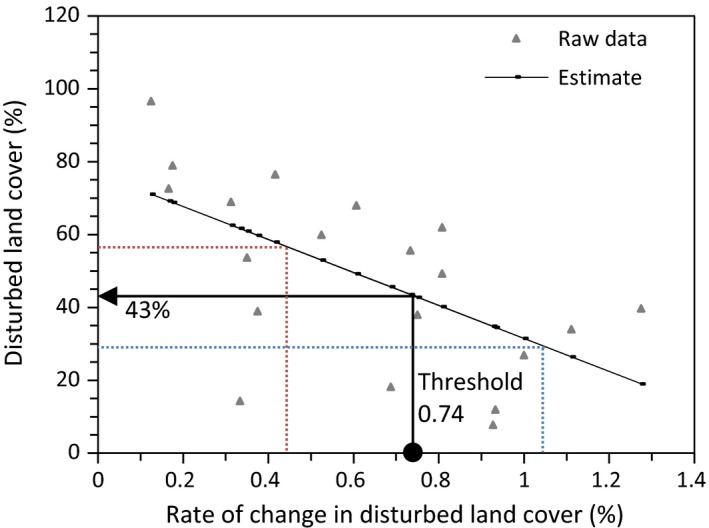
The amount of disturbed land cover in relation to the rate of change in disturbed land cover per WMU in the Alberta boreal forest (Pearson, *N* = 20, *R* = −.61, *p* < 0.005). The threshold between increasing and decreasing moose density (0.74% rate of change in disturbed land cover) corresponds to approximately 43% (range 29–56%) disturbed land cover

The threshold between decreasing and increasing moose density occurred at 0.74% new land disturbance per year (Figure 3). This rate of disturbance occurred at about 43% of disturbed land cover and the confidence limits indicated a range of 29%–56% of disturbed land cover within which moose population growth shifted from negative to positive (Figure 6). When land cover was already highly disturbed, there was a lower rate of disturbance (Figure 6). At lower rates of land cover disturbance, rate of change in moose density was increasing (Figure 3).

## 4 Discussion

Our results indicate that, in response to incremental disturbance, moose density declined with increasing land cover disturbance in landscapes with <43% disturbance. In these landscapes, moose density declines were associated with high rates of increase in linear disturbance which, in turn, were associated with increased wolf harvest. By contrast, in landscapes with >43% disturbance moose densities increased with additional disturbance while wolf harvest appeared to decline (Figure 5). This supports our prediction that moose and wolves show an inverse response to incremental landscape change and that there appears to be a shift in their response at about 43% of the landscape disturbed.

High wolf harvest in Alberta indexes high wolf populations (Robichaud & Boyce, 2010). Given that wolves are some of the main predators of moose affecting moose population growth (Ballard et al., 1987; Kuzyk, 2002; Messier, 1991), we suggest that high wolf densities reflect high predation pressure and that predation pressure peaks at intermediate linear densities.

Latombe et al. (2014) indicated that wolf presence appears to solicit complex anti‐predator responses showing that moose avoid areas frequented by wolves. Although our data do not show predation on moose per se, the results by Latombe et al. (2014) showing that moose respond inversely to the presence of wolves is supported by our findings on a landscape scale. Wolf predation is a main driver of moose populations as Messier (1991, 1994) showed that under high wolf densities, moose population growth is limited, while under low wolf densities, moose can reach carrying capacity at which point they compete for food.

Several studies found that, in largely forested areas, linear corridors facilitate the mobility of predators and their hunting success (Dickie et al., 2016; Kunkel & Pletscher, 2000; Latham et al., 2011). Whittington et al. (2005) found similar results but showed that wolves benefit from linear disturbance only up to a linear corridor density of 1.0 km/km^2^ and that wolves avoid areas with higher linear densities. By contrast, in highly disturbed areas where wolf populations decline, moose densities can increase to reach carrying capacity. Indeed, large prey, such as moose, often seek safety from predation in the vicinity of human activity (Hebblewhite et al., 2005; Muhly et al., 2011), and moose appear to thrive near urban areas and in landscapes with a high density of development (Maier et al., 2005; Schneider & Wasel, 2000; Torres et al., 2011). It appears that in such highly fragmented landscapes forage may be more available than in predominantly forested areas (Schneider & Wasel, 2000). This corresponds to our finding that having a positive rate of change in moose density is influenced by the amount of disturbed land cover. Therefore, it appears that the responses of the predator–prey system to incremental disturbance are qualitatively different in landscapes with low versus landscapes with high amounts of disturbance.

Some studies conclude that moose are resilient to landscape disturbance (Schneider & Wasel, 2000; Torres et al., 2011) while others found that moose are susceptible to it (Kunkel & Pletscher, 2000; Laurian et al., 2008; Stewart & Komers, 2012). Unfortunately, however, the above studies did not measure the amount or rate of land cover disturbed in their study areas. We can therefore only speculate, based on our findings, that these apparently contradictory conclusions about moose resilience to disturbance may both be correct, but that resilience appears to be dependent on the context of landscape degradation.

It appears that changes in predator–prey ratios affect ecosystem processes via trophic cascades (Estes et al., 2011; Ford et al., 2014); therefore, the inversely related changes in wolf and moose densities observed in our study could have consequences for ecosystem processes, such as the accumulation of plant biomass or changes to plant species richness (Estes et al., 2011; Hebblewhite et al., 2005). If this occurs in our study area, then the shift in the variables that we were able to measure may indicate a critical transition of the landscape to an alternate ecosystem state between 29% and 56% land cover disturbed (Figure 6).

Landscapes with >43% (the range being 29% to 56%) disturbance may offer more limited conservation options than less disturbed landscapes, because species compositions and ecosystem processes may be altered compared to their original state. While species responses to progressive habitat loss indicate a large range of species specificity, many species do not appear to be viable when more than about 50% of habitat is lost (Swift & Hannon, 2010). Indeed, predators in our study area seem to be virtually absent from landscapes with linear corridor density >1.0 km^2^ (Figure 5).

Our findings that a predator–prey system differs before and after a 43% threshold support the recommendation that novel ecosystems require novel approaches to conservation (Hobbs, Higgs, & Harris, 2009). We suggest that in highly disturbed landscapes, conservation decisions may need to focus on certain species and processes that can still exist in those conditions, particularly if incremental disturbance is expected in the future. Conservation decisions need to take into consideration both the amount and the rate of land cover disturbance. For example, land sparing, which promotes the setting aside of protected areas for biodiversity conservation while allowing “production areas” to maximize agricultural yield and other anthropogenic activities (Packer et al., 2013; Phalan, Onial, Balmford, & Green, 2011), may be the more effective strategy in highly disturbed regions. Land sharing, by contrast, promotes the re‐establishment of biodiversity outside of protected areas in human dominated landscapes and has been advocated as a sound approach to biodiversity conservation (Fischer et al., 2011; Mendenhall, 2011). However, we think that land sharing in highly disturbed landscapes would likely have limited success because system processes and the likely resulting species compositions may represent new ecosystems that are under different sets of controls compared to the original ones, differing in their response to incremental landscape change. We hypothesize that land sharing may be a useful conservation approach in relatively little disturbed landscapes, before a shift to novel ecosystems occurs, while land sparing may be the necessary approach in highly disturbed ones.

Our results support the call for the protection of at least 50% of a boreal forest region (International Boreal Conservation Science Panel 2013; Noss et al., 2012). Anthropogenic changes to land cover may result in the exceedance of ecological thresholds that may not be reversible (Groffman et al., 2006). We do not know whether the progression of disturbance in our study region is potentially reversible, but given the projected increase in industrial development (Government of Alberta 2012) and the near impossibility of re‐establishment to predisturbance conditions of certain habitat types (particularly of various wetland types, Rooney, Bayley, & Schindler, 2012), it is prudent to assume that, for conservation planning purposes, the landscape change in the Alberta boreal forest is permanent.

In our study area, the rate of land cover change based on Landsat image analysis was 0.8% per year (Komers & Stanojevic, 2013), which was comparable to the rates found by Potapov et al. (2011; 0.3% forest cover loss per year) and Hansen, Stehman, and Potapov (2010; 0.6% forest cover loss per year). Given these rates and amounts of disturbance, several of the WMUs that were <43% disturbed will reach the 50% threshold, called for by Noss et al. (2012) and the International Boreal Conservation Science Panel (2013), in 10 years or less (after our data collection ended in 2009). In such landscapes, there may not be sufficient time to develop and implement conservation strategies before the ecosystems change in their responses to progressive disturbance. We conclude that conservation decisions, such as, for example, land sharing versus land sparing strategies, need to be adapted not only to the amounts and but also to the rates of disturbance because conservation actions need to be prioritized in light of the impending ecosystem shifts.

## Conflict of Interest

None declared.
